# Supplementary material for: End Sequence Analysis Toolkit (ESAT) expands the extractable information from single-cell RNA-seq data
Source: Genome Res. 2016 Oct;26(10):1397–410. doi: 10.1101/gr.207902.116 (PMC5052061; doi:10.1101/gr.207902.116)
Supplement: Supplemental Material [file supp_gr.207902.116_Supp_Materials.tar.gz › ESAT-esat_v0.1/doc/html/classbroad_1_1core_1_1annotation_1_1_annotation_reader_3_01_t_01extends_01_genomic_annotation_01_4.html]

ESAT: broad.core.annotation.AnnotationReader< T extends GenomicAnnotation > Class Reference


ESAT


- Main Page
- Related Pages
- Packages
- Classes
- Files

- Class List
- Class Index
- Class Hierarchy
- Class Members

All Classes Namespaces Files Functions Variables Enumerator Pages

- broad
- core
- annotation
- AnnotationReader< T extends GenomicAnnotation >

Classes |
Public Member Functions |
Static Public Member Functions |
Protected Member Functions |
List of all members

broad.core.annotation.AnnotationReader< T extends GenomicAnnotation > Class Referenceabstract

|  |  |
| --- | --- |
| Classes | |
| class | **AnnotationSet< T extends LightweightGenomicAnnotation >** |
|  | |
| class | **trivialFilter** |
|  | |

|  |  |
| --- | --- |
| Public Member Functions | |
|  | AnnotationReader () |
|  | |
| void | init () |
|  | |
|  | AnnotationReader (Collection< T > annotations) |
|  | |
| void | clear () |
|  | |
| void | load (File source, AnnotationFactory<?extends T > factory) throws IOException, ParseException |
|  | |
| void | load (BufferedReader br, AnnotationFactory<?extends T > factory) throws IOException, ParseException |
|  | |
| void | load (File source, AnnotationFactory<?extends T > factory, GenomicAnnotationFilter< T > filter) throws IOException, ParseException |
|  | |
| void | load (BufferedReader br, AnnotationFactory<?extends T > factory, GenomicAnnotationFilter< T > filter) throws IOException, ParseException |
|  | |
| abstract int | parse (String file, GenomicAnnotationFilter< T > filter, AnnotationHandler handler) throws ParseException, IOException |
|  | |
| abstract int | parse (BufferedReader br, GenomicAnnotationFilter< T > filter, AnnotationHandler handler) throws ParseException, IOException |
|  | |
| Map< String, List< T > > | getChromosomeAnnotationMap () |
|  | |
| Iterator< String > | getChromosomeIterator () |
|  | |
| ShortBEDReader | cloneRegions () |
|  | |
| Map< String, List< T > > | getChromosomeAnnotationMap (int i) |
|  | |
| int | getBaseCoverage (List< T > annotations) |
|  | |
| int | numberCovered (List<?extends GenomicAnnotation > annotations) |
|  | |
| void | intersect (AnnotationReader< T > other) |
|  | |
| void | intersect (List<?extends GenomicAnnotation > annotations) |
|  | |
| Collection< T > | takeIntersection (List<?extends GenomicAnnotation > annotations) |
|  | |
| T | findClosest (final LightweightGenomicAnnotation annotation) |
|  | |
| T | findClosestNonOverlapping (final LightweightGenomicAnnotation annotation) |
|  | |
| T | findClosest (final LightweightGenomicAnnotation annotation, boolean nonOverlapping) |
|  | |
| Map< T,   LightweightGenomicAnnotation > | findClosestForAll (final AnnotationReader<?extends GenomicAnnotation > otherReader, boolean nonOverlapping) |
|  | |
| Map< T,   LightweightGenomicAnnotation > | findClosestForAll (final AnnotationReader<?extends GenomicAnnotation > otherReader) |
|  | |
| Map< T,   LightweightGenomicAnnotation > | findClosestNonoverlappingForAll (final AnnotationReader<?extends GenomicAnnotation > otherReader) |
|  | |
| int | getDistanceToClosest (final LightweightGenomicAnnotation annotation) |
|  | |
| Map< T, Integer > | getDistancesToClosest (final AnnotationReader<?extends GenomicAnnotation > otherReader) |
|  | |
| void | permuteAnnotationsOnChromosome (final Map< String, Integer > sizes) |
|  | |
| BasicAnnotationReader | takeComplement (List<?extends Annotation > annotations) |
|  | |
| List< Annotation > | getOverlappers (Annotation a) |
|  | |
| void | minus (List<?extends GenomicAnnotation > annotations) |
|  | |
| void | filterByOverlap (List<?extends Annotation > annotations) |
|  | |
| void | filterByOverlap (List<?extends Annotation > annotations, int extensionFactor) |
|  | |
| void | filterByOverlapOfLastExonOrAfterLastExon (List<?extends GenomicAnnotation > annotations, int extensionFactor) |
|  | |
| void | filterByOverlapOfLastExon (List<?extends GenomicAnnotation > annotations, int extensionFactor) |
|  | |
| void | filterByOverlapAndOrientation (List<?extends GenomicAnnotation > annotations) |
|  | |
| void | IncrementScoreIfOverlap (AnnotationReader<?extends GenomicAnnotation > annotReader, int setNumber, Boolean setToZero) |
|  | |
| int | getBaseCoverage () |
|  | |
| abstract T | createAnnotation (GenomicAnnotation a) |
|  | |
| void | shift (int amountToShift) |
|  | |
| void | merge () |
|  | |
| List< Annotation > | getOverlappers (List<?extends Annotation > annotationList) |
|  | |
| List< Annotation > | getOverlappers (List<?extends Annotation > annotationList, boolean fullyContained) |
|  | |
| List< Annotation > | getOverlappers (AnnotationReader<?extends GenomicAnnotation > other) |
|  | |
| int | getBasesCovered (Annotation region) |
|  | |
| int | getBasesCovered (Annotation region, boolean fullyContained) |
|  | |
| int | getBasesCovered (Annotation region, int fudgeFactor) |
|  | |
| int | getBasesCovered (Annotation region, int fudgeFactor, boolean fullyContained) |
|  | |
| List< T > | getOverlappers (LightweightGenomicAnnotation annotation) |
|  | |
| double | getScoreSum (LightweightGenomicAnnotation region) |
|  | |
| double | getAverageScore (LightweightGenomicAnnotation region) |
|  | |
| void | merge (int setNumber) |
|  | |
| void | concatAnnotationSet (int setNumber, List<?extends GenomicAnnotation > list) |
|  | |
| void | addAnnotation (T annotation, int track) |
|  | |
| void | addAnnotation (T annotation) |
|  | |
| void | addAnnotationToLastTrack (T annotation) |
|  | |
| List< T > | getAnnotationsForSequence (String sequenceName) |
|  | |
| List< T > | getAnnotationsForSequence (String sequenceName, int setNumber) |
|  | |
| Map< String, List< T > > | getSequenceAnnotationMap () |
|  | |
| Map< String, List< T > > | getSequenceAnnotationMap (int setNumber) |
|  | |
| void | addBrowserLine (String line) |
|  | |
| void | startTrack (String line) |
|  | |
| void | startTrack (String line, int trackNum) |
|  | |
| IntervalTree< T > | getChromosomeTree (String chr, int i) |
|  | |
| IntervalTree< T > | getChromosomeTree (String chr) |
|  | |
| Map< String, IntervalTree< T > > | getChromosomeTreeMap (int i) |
|  | |
| Map< String, IntervalTree< T > > | getChromosomeTreeMap () |
|  | |
| String | getTrackInfo (int i) |
|  | |
| List< T > | getChromosomeBEDs (String chr) |
|  | |
| List< T > | getChromosomeBEDs (String chr, int i) |
|  | |
| List< T > | getAnnotationList (int setNumber) |
|  | |
| T | getAnnotation (int setNumber, String name) |
|  | |
| boolean | containsAnnotation (int setNumber, String name) |
|  | |
| T | getAnnotation (String name) |
|  | |
| boolean | containsAnnotation (String name) |
|  | |
| void | shuffleScores (String chr, int setNum) |
|  | |
| void | shuffleScores (String chr) |
|  | |
| List< T > | getAnnotationList () |
|  | |
| int | numOfAnnotation (int setNum) |
|  | |
| int | size () |
|  | |
| String | toStringForWriting () |
|  | |
| void | write (BufferedWriter bw) throws IOException |
|  | |
| void | write (String outputFile) throws IOException |
|  | |
| void | filterByScore (double set1FilterScore) |
|  | |
| void | addFilter (Predicate< T > filter) |
|  | |
| void | addFilters (Collection< Predicate< T >> filters) |
|  | |

|  |  |
| --- | --- |
| Static Public Member Functions | |
| static Map< Annotation, Integer > | getLastExons (List<?extends Annotation > annotations) |
|  | |

|  |  |
| --- | --- |
| Protected Member Functions | |
| int | parse (String file, AnnotationFactory<?extends T > factory, GenomicAnnotationFilter< T > filter, AnnotationHandler handler) throws ParseException, IOException |
|  | |
| int | parse (BufferedReader br, AnnotationFactory<?extends T > factory, GenomicAnnotationFilter< T > filter, AnnotationHandler handler) throws ParseException |
|  | |
| void | addAnnotationSet (AnnotationSet< T > track) |
|  | |

## Constructor & Destructor Documentation

|  |  |  |  |  |
| --- | --- | --- | --- | --- |
| broad.core.annotation.AnnotationReader< T extends GenomicAnnotation >.AnnotationReader | ( |  | ) |  |

|  |  |  |  |  |  |
| --- | --- | --- | --- | --- | --- |
| broad.core.annotation.AnnotationReader< T extends GenomicAnnotation >.AnnotationReader | ( | Collection< T > | *annotations* | ) |  |

Jesse Engreitz September 26, 2012 Allow for creating a reader from a collection of annotations

Parameters
:   |  |  |
    | --- | --- |
    | annotations |  |

## Member Function Documentation

|  |  |  |  |
| --- | --- | --- | --- |
| void broad.core.annotation.AnnotationReader< T extends GenomicAnnotation >.addAnnotation | ( | T | *annotation*, |
|  |  | int | *track* |
|  | ) |  |  |

|  |  |  |  |  |  |
| --- | --- | --- | --- | --- | --- |
| void broad.core.annotation.AnnotationReader< T extends GenomicAnnotation >.addAnnotation | ( | T | *annotation* | ) |  |

Here is the call graph for this function:

Here is the caller graph for this function:

|  |  |  |  |  |  |
| --- | --- | --- | --- | --- | --- |
| void broad.core.annotation.AnnotationReader< T extends GenomicAnnotation >.addAnnotationSet | ( | AnnotationSet< T > | *track* | ) |  |

protected

|  |  |  |  |  |  |
| --- | --- | --- | --- | --- | --- |
| void broad.core.annotation.AnnotationReader< T extends GenomicAnnotation >.addAnnotationToLastTrack | ( | T | *annotation* | ) |  |

|  |  |  |  |  |  |
| --- | --- | --- | --- | --- | --- |
| void broad.core.annotation.AnnotationReader< T extends GenomicAnnotation >.addBrowserLine | ( | String | *line* | ) |  |

|  |  |  |  |  |  |
| --- | --- | --- | --- | --- | --- |
| void broad.core.annotation.AnnotationReader< T extends GenomicAnnotation >.addFilter | ( | Predicate< T > | *filter* | ) |  |

|  |  |  |  |  |  |
| --- | --- | --- | --- | --- | --- |
| void broad.core.annotation.AnnotationReader< T extends GenomicAnnotation >.addFilters | ( | Collection< Predicate< T >> | *filters* | ) |  |

|  |  |  |  |  |
| --- | --- | --- | --- | --- |
| void broad.core.annotation.AnnotationReader< T extends GenomicAnnotation >.clear | ( |  | ) |  |

|  |  |  |  |  |
| --- | --- | --- | --- | --- |
| ShortBEDReader broad.core.annotation.AnnotationReader< T extends GenomicAnnotation >.cloneRegions | ( |  | ) |  |

|  |  |  |  |
| --- | --- | --- | --- |
| void broad.core.annotation.AnnotationReader< T extends GenomicAnnotation >.concatAnnotationSet | ( | int | *setNumber*, |
|  |  | List<?extends GenomicAnnotation > | *list* |
|  | ) |  |  |

Moran, Feb22 2010 concat an annotation set to an exciting one (without merging) WARNING: assumes that same types are concatenated- uses casting to current objets annotation type

|  |  |  |  |
| --- | --- | --- | --- |
| boolean broad.core.annotation.AnnotationReader< T extends GenomicAnnotation >.containsAnnotation | ( | int | *setNumber*, |
|  |  | String | *name* |
|  | ) |  |  |

|  |  |  |  |  |  |
| --- | --- | --- | --- | --- | --- |
| boolean broad.core.annotation.AnnotationReader< T extends GenomicAnnotation >.containsAnnotation | ( | String | *name* | ) |  |

|  |  |  |  |  |  |
| --- | --- | --- | --- | --- | --- |
| abstract T broad.core.annotation.AnnotationReader< T extends GenomicAnnotation >.createAnnotation | ( | GenomicAnnotation | *a* | ) |  |

abstract

|  |  |  |  |  |  |
| --- | --- | --- | --- | --- | --- |
| void broad.core.annotation.AnnotationReader< T extends GenomicAnnotation >.filterByOverlap | ( | List<?extends Annotation > | *annotations* | ) |  |

Filters this reader by removing all elements that do not overlap the given list of annotations

Parameters
:   |  |  |
    | --- | --- |
    | annotations | a list of Annotations to filter |

|  |  |  |  |
| --- | --- | --- | --- |
| void broad.core.annotation.AnnotationReader< T extends GenomicAnnotation >.filterByOverlap | ( | List<?extends Annotation > | *annotations*, |
|  |  | int | *extensionFactor* |
|  | ) |  |  |

Here is the call graph for this function:

|  |  |  |  |  |  |
| --- | --- | --- | --- | --- | --- |
| void broad.core.annotation.AnnotationReader< T extends GenomicAnnotation >.filterByOverlapAndOrientation | ( | List<?extends GenomicAnnotation > | *annotations* | ) |  |

ADDED BY MORAN Feb 8th 2010 Filters this reader by removing all elements that do not overlap AND HAVE THE SAME ORIENTATION as the given list of annotations

Parameters
:   |  |  |
    | --- | --- |
    | annotations | a list of Annotations to filter |

Here is the call graph for this function:

|  |  |  |  |
| --- | --- | --- | --- |
| void broad.core.annotation.AnnotationReader< T extends GenomicAnnotation >.filterByOverlapOfLastExon | ( | List<?extends GenomicAnnotation > | *annotations*, |
|  |  | int | *extensionFactor* |
|  | ) |  |  |

Here is the call graph for this function:

|  |  |  |  |
| --- | --- | --- | --- |
| void broad.core.annotation.AnnotationReader< T extends GenomicAnnotation >.filterByOverlapOfLastExonOrAfterLastExon | ( | List<?extends GenomicAnnotation > | *annotations*, |
|  |  | int | *extensionFactor* |
|  | ) |  |  |

Here is the call graph for this function:

|  |  |  |  |  |  |
| --- | --- | --- | --- | --- | --- |
| void broad.core.annotation.AnnotationReader< T extends GenomicAnnotation >.filterByScore | ( | double | *set1FilterScore* | ) |  |

|  |  |  |  |  |  |
| --- | --- | --- | --- | --- | --- |
| T broad.core.annotation.AnnotationReader< T extends GenomicAnnotation >.findClosest | ( | final LightweightGenomicAnnotation | *annotation* | ) |  |

|  |  |  |  |
| --- | --- | --- | --- |
| T broad.core.annotation.AnnotationReader< T extends GenomicAnnotation >.findClosest | ( | final LightweightGenomicAnnotation | *annotation*, |
|  |  | boolean | *nonOverlapping* |
|  | ) |  |  |

Added "nonOverlapping" option June 13, 2012

Parameters
:   |  |  |
    | --- | --- |
    | nonOverlapping | Set to true to get the closest non-overlapping annotation |

Here is the call graph for this function:

|  |  |  |  |
| --- | --- | --- | --- |
| Map<T,LightweightGenomicAnnotation> broad.core.annotation.AnnotationReader< T extends GenomicAnnotation >.findClosestForAll | ( | final AnnotationReader<?extends GenomicAnnotation > | *otherReader*, |
|  |  | boolean | *nonOverlapping* |
|  | ) |  |  |

Jesse Engreitz June 24, 2012 Return a map of elements in this reader to the closest elements in another reader

Parameters
:   |  |  |
    | --- | --- |
    | otherReader | second set of annotations |
    | nonOverlapping | set to true to get the closest non-overlapping elements |

Returns

|  |  |  |  |  |  |
| --- | --- | --- | --- | --- | --- |
| Map<T,LightweightGenomicAnnotation> broad.core.annotation.AnnotationReader< T extends GenomicAnnotation >.findClosestForAll | ( | final AnnotationReader<?extends GenomicAnnotation > | *otherReader* | ) |  |

Here is the call graph for this function:

Here is the caller graph for this function:

|  |  |  |  |  |  |
| --- | --- | --- | --- | --- | --- |
| T broad.core.annotation.AnnotationReader< T extends GenomicAnnotation >.findClosestNonOverlapping | ( | final LightweightGenomicAnnotation | *annotation* | ) |  |

|  |  |  |  |  |  |
| --- | --- | --- | --- | --- | --- |
| Map<T,LightweightGenomicAnnotation> broad.core.annotation.AnnotationReader< T extends GenomicAnnotation >.findClosestNonoverlappingForAll | ( | final AnnotationReader<?extends GenomicAnnotation > | *otherReader* | ) |  |

|  |  |  |  |
| --- | --- | --- | --- |
| T broad.core.annotation.AnnotationReader< T extends GenomicAnnotation >.getAnnotation | ( | int | *setNumber*, |
|  |  | String | *name* |
|  | ) |  |  |

|  |  |  |  |  |  |
| --- | --- | --- | --- | --- | --- |
| T broad.core.annotation.AnnotationReader< T extends GenomicAnnotation >.getAnnotation | ( | String | *name* | ) |  |

|  |  |  |  |  |  |
| --- | --- | --- | --- | --- | --- |
| List<T> broad.core.annotation.AnnotationReader< T extends GenomicAnnotation >.getAnnotationList | ( | int | *setNumber* | ) |  |

|  |  |  |  |  |
| --- | --- | --- | --- | --- |
| List<T> broad.core.annotation.AnnotationReader< T extends GenomicAnnotation >.getAnnotationList | ( |  | ) |  |

Here is the call graph for this function:

Here is the caller graph for this function:

|  |  |  |  |  |  |
| --- | --- | --- | --- | --- | --- |
| List<T> broad.core.annotation.AnnotationReader< T extends GenomicAnnotation >.getAnnotationsForSequence | ( | String | *sequenceName* | ) |  |

|  |  |  |  |
| --- | --- | --- | --- |
| List<T> broad.core.annotation.AnnotationReader< T extends GenomicAnnotation >.getAnnotationsForSequence | ( | String | *sequenceName*, |
|  |  | int | *setNumber* |
|  | ) |  |  |

|  |  |  |  |  |  |
| --- | --- | --- | --- | --- | --- |
| double broad.core.annotation.AnnotationReader< T extends GenomicAnnotation >.getAverageScore | ( | LightweightGenomicAnnotation | *region* | ) |  |

|  |  |  |  |  |  |
| --- | --- | --- | --- | --- | --- |
| int broad.core.annotation.AnnotationReader< T extends GenomicAnnotation >.getBaseCoverage | ( | List< T > | *annotations* | ) |  |

|  |  |  |  |  |
| --- | --- | --- | --- | --- |
| int broad.core.annotation.AnnotationReader< T extends GenomicAnnotation >.getBaseCoverage | ( |  | ) |  |

Here is the call graph for this function:

Here is the caller graph for this function:

|  |  |  |  |  |  |
| --- | --- | --- | --- | --- | --- |
| int broad.core.annotation.AnnotationReader< T extends GenomicAnnotation >.getBasesCovered | ( | Annotation | *region* | ) |  |

Adds a 0 fudge fact

Parameters
:   |  |  |
    | --- | --- |
    | region |  |

Returns

Exceptions
:   |  |  |
    | --- | --- |
    | IOException |  |

|  |  |  |  |
| --- | --- | --- | --- |
| int broad.core.annotation.AnnotationReader< T extends GenomicAnnotation >.getBasesCovered | ( | Annotation | *region*, |
|  |  | boolean | *fullyContained* |
|  | ) |  |  |

|  |  |  |  |
| --- | --- | --- | --- |
| int broad.core.annotation.AnnotationReader< T extends GenomicAnnotation >.getBasesCovered | ( | Annotation | *region*, |
|  |  | int | *fudgeFactor* |
|  | ) |  |  |

|  |  |  |  |
| --- | --- | --- | --- |
| int broad.core.annotation.AnnotationReader< T extends GenomicAnnotation >.getBasesCovered | ( | Annotation | *region*, |
|  |  | int | *fudgeFactor*, |
|  |  | boolean | *fullyContained* |
|  | ) |  |  |

Here is the call graph for this function:

|  |  |  |  |  |
| --- | --- | --- | --- | --- |
| Map<String, List<T> > broad.core.annotation.AnnotationReader< T extends GenomicAnnotation >.getChromosomeAnnotationMap | ( |  | ) |  |

|  |  |  |  |  |  |
| --- | --- | --- | --- | --- | --- |
| Map<String, List<T> > broad.core.annotation.AnnotationReader< T extends GenomicAnnotation >.getChromosomeAnnotationMap | ( | int | *i* | ) |  |

|  |  |  |  |  |  |
| --- | --- | --- | --- | --- | --- |
| List<T> broad.core.annotation.AnnotationReader< T extends GenomicAnnotation >.getChromosomeBEDs | ( | String | *chr* | ) |  |

|  |  |  |  |
| --- | --- | --- | --- |
| List<T> broad.core.annotation.AnnotationReader< T extends GenomicAnnotation >.getChromosomeBEDs | ( | String | *chr*, |
|  |  | int | *i* |
|  | ) |  |  |

|  |  |  |  |  |
| --- | --- | --- | --- | --- |
| Iterator<String> broad.core.annotation.AnnotationReader< T extends GenomicAnnotation >.getChromosomeIterator | ( |  | ) |  |

|  |  |  |  |
| --- | --- | --- | --- |
| IntervalTree<T> broad.core.annotation.AnnotationReader< T extends GenomicAnnotation >.getChromosomeTree | ( | String | *chr*, |
|  |  | int | *i* |
|  | ) |  |  |

|  |  |  |  |  |  |
| --- | --- | --- | --- | --- | --- |
| IntervalTree<T> broad.core.annotation.AnnotationReader< T extends GenomicAnnotation >.getChromosomeTree | ( | String | *chr* | ) |  |

|  |  |  |  |  |  |
| --- | --- | --- | --- | --- | --- |
| Map<String,IntervalTree<T> > broad.core.annotation.AnnotationReader< T extends GenomicAnnotation >.getChromosomeTreeMap | ( | int | *i* | ) |  |

|  |  |  |  |  |
| --- | --- | --- | --- | --- |
| Map<String,IntervalTree<T> > broad.core.annotation.AnnotationReader< T extends GenomicAnnotation >.getChromosomeTreeMap | ( |  | ) |  |

|  |  |  |  |  |  |
| --- | --- | --- | --- | --- | --- |
| Map<T, Integer> broad.core.annotation.AnnotationReader< T extends GenomicAnnotation >.getDistancesToClosest | ( | final AnnotationReader<?extends GenomicAnnotation > | *otherReader* | ) |  |

|  |  |  |  |  |  |
| --- | --- | --- | --- | --- | --- |
| int broad.core.annotation.AnnotationReader< T extends GenomicAnnotation >.getDistanceToClosest | ( | final LightweightGenomicAnnotation | *annotation* | ) |  |

Jesse - June 13, 2012

Parameters
:   |  |  |
    | --- | --- |
    | annotation |  |

Returns

|  |  |  |  |  |  |
| --- | --- | --- | --- | --- | --- |
| static Map<Annotation, Integer> broad.core.annotation.AnnotationReader< T extends GenomicAnnotation >.getLastExons | ( | List<?extends Annotation > | *annotations* | ) |  |

static

Here is the call graph for this function:

|  |  |  |  |  |  |
| --- | --- | --- | --- | --- | --- |
| List<Annotation> broad.core.annotation.AnnotationReader< T extends GenomicAnnotation >.getOverlappers | ( | Annotation | *a* | ) |  |

|  |  |  |  |  |  |
| --- | --- | --- | --- | --- | --- |
| List<Annotation> broad.core.annotation.AnnotationReader< T extends GenomicAnnotation >.getOverlappers | ( | List<?extends Annotation > | *annotationList* | ) |  |

Gets a list of overlapping annotations

|  |  |  |  |
| --- | --- | --- | --- |
| List<Annotation> broad.core.annotation.AnnotationReader< T extends GenomicAnnotation >.getOverlappers | ( | List<?extends Annotation > | *annotationList*, |
|  |  | boolean | *fullyContained* |
|  | ) |  |  |

Here is the call graph for this function:

|  |  |  |  |  |  |
| --- | --- | --- | --- | --- | --- |
| List<Annotation> broad.core.annotation.AnnotationReader< T extends GenomicAnnotation >.getOverlappers | ( | AnnotationReader<?extends GenomicAnnotation > | *other* | ) |  |

|  |  |  |  |  |  |
| --- | --- | --- | --- | --- | --- |
| List<T> broad.core.annotation.AnnotationReader< T extends GenomicAnnotation >.getOverlappers | ( | LightweightGenomicAnnotation | *annotation* | ) |  |

Gets a list of overlapping annotations

public Collection<T> getOverlappers(AnnotationReader<T> other) { Set<T> overlappers = new TreeSet<T>(); Iterator<String> chrIt = other.getChromosomeIterator(); while(chrIt.hasNext()) { String chr = chrIt.next(); Iterator<T> annotationIt = other.getChromosomeTree(chr).valueIterator(); IntervalTree<T> tree = getChromosomeTree(chr); if(tree == null) { continue; } while(annotationIt.hasNext()) { LightweightGenomicAnnotation annot = annotationIt.next(); Iterator<T> overlapperIt = new IntervalTree.ValuesIterator<T>(tree.overlappers(annot.getStart(), annot.getEnd())); while(overlapperIt.hasNext()) { T overlapper = overlapperIt.next(); overlappers.add(overlapper); } } } return overlappers; }

Here is the call graph for this function:

|  |  |  |  |  |  |
| --- | --- | --- | --- | --- | --- |
| double broad.core.annotation.AnnotationReader< T extends GenomicAnnotation >.getScoreSum | ( | LightweightGenomicAnnotation | *region* | ) |  |

|  |  |  |  |  |
| --- | --- | --- | --- | --- |
| Map<String, List<T> > broad.core.annotation.AnnotationReader< T extends GenomicAnnotation >.getSequenceAnnotationMap | ( |  | ) |  |

|  |  |  |  |  |  |
| --- | --- | --- | --- | --- | --- |
| Map<String, List<T> > broad.core.annotation.AnnotationReader< T extends GenomicAnnotation >.getSequenceAnnotationMap | ( | int | *setNumber* | ) |  |

|  |  |  |  |  |  |
| --- | --- | --- | --- | --- | --- |
| String broad.core.annotation.AnnotationReader< T extends GenomicAnnotation >.getTrackInfo | ( | int | *i* | ) |  |

|  |  |  |  |
| --- | --- | --- | --- |
| void broad.core.annotation.AnnotationReader< T extends GenomicAnnotation >.IncrementScoreIfOverlap | ( | AnnotationReader<?extends GenomicAnnotation > | *annotReader*, |
|  |  | int | *setNumber*, |
|  |  | Boolean | *setToZero* |
|  | ) |  |  |

Moran FEB23 2010 Increments by one the score of the annotations in the current object if they overlap the input set. Set to zero flag will first initialize all scores to zero

Returns

|  |  |  |  |  |
| --- | --- | --- | --- | --- |
| void broad.core.annotation.AnnotationReader< T extends GenomicAnnotation >.init | ( |  | ) |  |

|  |  |  |  |  |  |
| --- | --- | --- | --- | --- | --- |
| void broad.core.annotation.AnnotationReader< T extends GenomicAnnotation >.intersect | ( | AnnotationReader< T > | *other* | ) |  |

|  |  |  |  |  |  |
| --- | --- | --- | --- | --- | --- |
| void broad.core.annotation.AnnotationReader< T extends GenomicAnnotation >.intersect | ( | List<?extends GenomicAnnotation > | *annotations* | ) |  |

|  |  |  |  |
| --- | --- | --- | --- |
| void broad.core.annotation.AnnotationReader< T extends GenomicAnnotation >.load | ( | File | *source*, |
|  |  | AnnotationFactory<?extends T > | *factory* |
|  | ) |  | throws IOException, ParseException |

|  |  |  |  |
| --- | --- | --- | --- |
| void broad.core.annotation.AnnotationReader< T extends GenomicAnnotation >.load | ( | BufferedReader | *br*, |
|  |  | AnnotationFactory<?extends T > | *factory* |
|  | ) |  | throws IOException, ParseException |

|  |  |  |  |
| --- | --- | --- | --- |
| void broad.core.annotation.AnnotationReader< T extends GenomicAnnotation >.load | ( | File | *source*, |
|  |  | AnnotationFactory<?extends T > | *factory*, |
|  |  | GenomicAnnotationFilter< T > | *filter* |
|  | ) |  | throws IOException, ParseException |

|  |  |  |  |
| --- | --- | --- | --- |
| void broad.core.annotation.AnnotationReader< T extends GenomicAnnotation >.load | ( | BufferedReader | *br*, |
|  |  | AnnotationFactory<?extends T > | *factory*, |
|  |  | GenomicAnnotationFilter< T > | *filter* |
|  | ) |  | throws IOException, ParseException |

|  |  |  |  |  |
| --- | --- | --- | --- | --- |
| void broad.core.annotation.AnnotationReader< T extends GenomicAnnotation >.merge | ( |  | ) |  |

Merge list of annotations by combining overlapping annotations into one.

Here is the call graph for this function:

Here is the caller graph for this function:

|  |  |  |  |  |  |
| --- | --- | --- | --- | --- | --- |
| void broad.core.annotation.AnnotationReader< T extends GenomicAnnotation >.merge | ( | int | *setNumber* | ) |  |

Merge list of annotations by combining overlapping annotations into one.

|  |  |  |  |  |  |
| --- | --- | --- | --- | --- | --- |
| void broad.core.annotation.AnnotationReader< T extends GenomicAnnotation >.minus | ( | List<?extends GenomicAnnotation > | *annotations* | ) |  |

Removes from this reader all elements that overlap the given list.

Parameters
:   |  |  |
    | --- | --- |
    | annotations |  |

Here is the call graph for this function:

|  |  |  |  |  |  |
| --- | --- | --- | --- | --- | --- |
| int broad.core.annotation.AnnotationReader< T extends GenomicAnnotation >.numberCovered | ( | List<?extends GenomicAnnotation > | *annotations* | ) |  |

Here is the call graph for this function:

|  |  |  |  |  |  |
| --- | --- | --- | --- | --- | --- |
| int broad.core.annotation.AnnotationReader< T extends GenomicAnnotation >.numOfAnnotation | ( | int | *setNum* | ) |  |

|  |  |  |  |
| --- | --- | --- | --- |
| abstract int broad.core.annotation.AnnotationReader< T extends GenomicAnnotation >.parse | ( | String | *file*, |
|  |  | GenomicAnnotationFilter< T > | *filter*, |
|  |  | AnnotationHandler | *handler* |
|  | ) |  | throws ParseException, IOException |

abstract

|  |  |  |  |
| --- | --- | --- | --- |
| int broad.core.annotation.AnnotationReader< T extends GenomicAnnotation >.parse | ( | String | *file*, |
|  |  | AnnotationFactory<?extends T > | *factory*, |
|  |  | GenomicAnnotationFilter< T > | *filter*, |
|  |  | AnnotationHandler | *handler* |
|  | ) |  | throws ParseException, IOException |

protected

|  |  |  |  |
| --- | --- | --- | --- |
| abstract int broad.core.annotation.AnnotationReader< T extends GenomicAnnotation >.parse | ( | BufferedReader | *br*, |
|  |  | GenomicAnnotationFilter< T > | *filter*, |
|  |  | AnnotationHandler | *handler* |
|  | ) |  | throws ParseException, IOException |

abstract

|  |  |  |  |
| --- | --- | --- | --- |
| int broad.core.annotation.AnnotationReader< T extends GenomicAnnotation >.parse | ( | BufferedReader | *br*, |
|  |  | AnnotationFactory<?extends T > | *factory*, |
|  |  | GenomicAnnotationFilter< T > | *filter*, |
|  |  | AnnotationHandler | *handler* |
|  | ) |  | throws ParseException |

protected

|  |  |  |  |  |  |
| --- | --- | --- | --- | --- | --- |
| void broad.core.annotation.AnnotationReader< T extends GenomicAnnotation >.permuteAnnotationsOnChromosome | ( | final Map< String, Integer > | *sizes* | ) |  |

Jesse Engreitz June 24, 2012 Takes the annotations in the reader and permutes them on their respective chromosomes. Note that this alters the positions of annotations in the reader, although not their sizes;

Parameters
:   |  |  |
    | --- | --- |
    | sizes | map of chromosome sizes |

|  |  |  |  |  |  |
| --- | --- | --- | --- | --- | --- |
| void broad.core.annotation.AnnotationReader< T extends GenomicAnnotation >.shift | ( | int | *amountToShift* | ) |  |

|  |  |  |  |
| --- | --- | --- | --- |
| void broad.core.annotation.AnnotationReader< T extends GenomicAnnotation >.shuffleScores | ( | String | *chr*, |
|  |  | int | *setNum* |
|  | ) |  |  |

|  |  |  |  |  |  |
| --- | --- | --- | --- | --- | --- |
| void broad.core.annotation.AnnotationReader< T extends GenomicAnnotation >.shuffleScores | ( | String | *chr* | ) |  |

|  |  |  |  |  |
| --- | --- | --- | --- | --- |
| int broad.core.annotation.AnnotationReader< T extends GenomicAnnotation >.size | ( |  | ) |  |

Jesse June 23, 2012

Returns
:   total number of annotations

|  |  |  |  |  |  |
| --- | --- | --- | --- | --- | --- |
| void broad.core.annotation.AnnotationReader< T extends GenomicAnnotation >.startTrack | ( | String | *line* | ) |  |

|  |  |  |  |
| --- | --- | --- | --- |
| void broad.core.annotation.AnnotationReader< T extends GenomicAnnotation >.startTrack | ( | String | *line*, |
|  |  | int | *trackNum* |
|  | ) |  |  |

|  |  |  |  |  |  |
| --- | --- | --- | --- | --- | --- |
| BasicAnnotationReader broad.core.annotation.AnnotationReader< T extends GenomicAnnotation >.takeComplement | ( | List<?extends Annotation > | *annotations* | ) |  |

Takes the complement in the given list in this Reader. That is, if an annotation in the list reader does not overlap any in the this reader, the annotation is kept intact. If it is contained in any annotation in the reader then it is removed. If it incompletely overlapped, then the annotation resulting from taking out overlapping regions replaces the overlapped one.

|  |  |  |  |  |  |
| --- | --- | --- | --- | --- | --- |
| Collection<T> broad.core.annotation.AnnotationReader< T extends GenomicAnnotation >.takeIntersection | ( | List<?extends GenomicAnnotation > | *annotations* | ) |  |

|  |  |  |  |  |
| --- | --- | --- | --- | --- |
| String broad.core.annotation.AnnotationReader< T extends GenomicAnnotation >.toStringForWriting | ( |  | ) |  |

Jesse June 23, 2012

Returns
:   string representation of all annotations, one per line

|  |  |  |  |  |  |
| --- | --- | --- | --- | --- | --- |
| void broad.core.annotation.AnnotationReader< T extends GenomicAnnotation >.write | ( | BufferedWriter | *bw* | ) | throws IOException |

Author
:   mgarber

Parameters
:   |  |  |
    | --- | --- |
    | BufferedWriter | - buffer to write annotations to |

Returns
:   writes all annotations to the provided BufferedWriter

Exceptions
:   |  |  |
    | --- | --- |
    | IOException |  |

|  |  |  |  |  |  |
| --- | --- | --- | --- | --- | --- |
| void broad.core.annotation.AnnotationReader< T extends GenomicAnnotation >.write | ( | String | *outputFile* | ) | throws IOException |

Author
:   mgarber

Parameters
:   |  |  |
    | --- | --- |
    | BufferedWriter | - buffer to write annotations to |

Returns
:   writes all annotations to the provided BufferedWriter

Exceptions
:   |  |  |
    | --- | --- |
    | IOException |  |

---

The documentation for this class was generated from the following file:

- java/broad/core/annotation/AnnotationReader.java


---

Generated on Fri Aug 15 2014 15:10:50 for ESAT by  

 1.8.7
